# Antineutrophil cytoplasmic antibody-associated vasculitis complicated by subarachnoid hemorrhage: A case report and literature review

**DOI:** 10.1097/MD.0000000000047227

**Published:** 2026-01-23

**Authors:** Ping Dong, Jie Li, Jing Li

**Affiliations:** a Department of Nephrology, Suzhou Research Center of Medical School, Suzhou Hospital, Affiliated Hospital of Medical School, Nanjing University, Suzhou, China; b Department of Neurology, Suzhou Research Center of Medical School, Suzhou Hospital, Affiliated Hospital of Medical School, Nanjing University, Suzhou, China.

**Keywords:** anterior communicating artery aneurysm, antineutrophil cytoplasmic antibody-associated vasculitis, case report, subarachnoid hemorrhage

## Abstract

**Rationale:**

antineutrophil cytoplasmic antibody (ANCA)-associated vasculitis (AAV) is an autoimmune syndrome characterized by necrotizing inflammation of small-to medium-sized vessels accompanied by the presence of peripheral blood ANCA. Subarachnoid hemorrhage (SAH) is an uncommon complication of AAV. The purpose of this report is to present a rare case of AAV complicated by SAH and to review the existing literature, thereby enhancing awareness of this severe neurological manifestation and its management strategies.

**Patient concerns:**

We report the case of a 56-year-old male who presented with cough, blood-tinged sputum, proteinuria, elevated serum creatinine levels, and headache. Laboratory examinations confirmed the presence of anti-myeloperoxidaseantibody, imaging revealed SAH, and angiography identified a ruptured anterior communicating artery aneurysm.

**Diagnoses:**

The final diagnosis was AAV complicated by SAH.

**Interventions:**

The patient underwent prompt endovascular coiling, with concurrent immunosuppression using methylprednisolone and intravenous cyclophosphamide.

**Outcomes:**

Following endovascular coiling, the patient’s subarachnoid hemorrhage and associated neurological symptoms (headache, dizziness) resolved completely. Immunosuppressive therapy led to the resolution of respiratory symptoms (cough and blood-tinged sputum), the clearance of hematuria, and a significant reduction in inflammatory markers, such as C-reactive protein (CRP) and erythrocyte sedimentation rate. However, despite immunosuppressive treatment, with serum creatinine levels fluctuating between 320 and 450 μmol/L, and heavy proteinuria (e.g., 24-hour urine protein of 3360.21mg) persisted. Additionally, the patient developed deep vein thrombosis 1 month after discharge, which was successfully managed with anticoagulation therapy and the placement of an inferior vena cava filter.

**Lessons:**

SAH is a rare but life-threatening complication of AAV. Vigilance for acute neurological symptoms, such as headache, enables early intervention. Combined endovascular repair for SAH and immunosuppressive therapy significantly improve outcomes.

## 1. Introduction

Antineutrophil cytoplasmic antibody (ANCA)-associated vasculitis (AAV) is a group of multisystem autoimmune diseases predominantly affecting small vessels^,[1,2]^ including capillaries, small veins, and small arteries.^[3]^ Clinically, AAV can be differentiated into granulomatosis with polyangiitis (GPA), microscopic polyangiitis (MPA), and eosinophilic GPA (EGPA).^[4]^ Proteinase 3 (PR3) and myeloperoxidase (MPO) have been identified as the primary target antigens.^[5]^ AAV has a notable prevalence in Caucasian and Asian populations. Systemic, pulmonary, and renal involvement are the most frequent manifestations, affecting approximately 50%, 50%, and 64% of patients, respectively.^[6]^ Although central nervous system (CNS) involvement is rare, with subarachnoid hemorrhage (SAH) being particularly uncommon, MPA may present with stroke, intracerebral hemorrhage, SAH, or spinal SAH.^[7–9]^

Herein, we report a rare case of AAV complicated by SAH.

## 2. Case report

A 56-year-old male presented to the Nephrology Department with a 2-week history of coughing and expectoration and a 1-week history of eyelid and bilateral foot edema. The patient reported no history of hypertension, diabetes, specific medication use, or trauma. He had smoked for more than 30 years at a rate of 10 to 15 cigarettes per day and denied any family history of cerebrovascular or cardiovascular diseases. The patient first experienced coughing and expectoration 2 weeks prior and had consulted the respiratory department a week earlier, where he was prescribed oral antibiotics (moxifloxacin hydrochloride tablets, 0.4 g po, qd); however, his symptoms did not improve. Subsequently, he developed eyelid and bilateral foot edema, and was thus referred to the Nephrology Department for further evaluation due to suspected renal involvement.

Upon this visit to the Nephrology Department, his blood pressure was 125/67 mm Hg, lung auscultation revealed coarse breath sounds with occasional moist rales in the lower lobes, and noticeable edema was observed in the eyelids and dorsum of both feet. Laboratory examination revealed a white blood cell count of 7.08 × 10^9^/L, neutrophils at 62.8%, hemoglobin at 105 g/L, platelets at 263 × 10^9^/L, and serum creatinine (SCr) at 344.6 µmol/L. Chest computed tomography (CT) showed interstitial changes in the lower lobe of the left lung and mucus plugging with bronchiectasis in the sublingual segment of the upper lobe of the left lung, necessitating hospitalization.

Upon admission, the patient’s blood pressure was 151/80 mm Hg, albumin (Alb) level was 32.7 g/L, urine protein level was 2+, and microscopic examination revealed 5 to 7 red blood cells per high-power field, SCr was 351 µmol/L, urine microalbumin/creatinine ratio was 390.92 mg/mmol, and complement C3 and C4 levels were normal. Renal ultrasonography showed normal-sized kidneys with increased parenchymal echogenicity and preserved corticomedullary differentiation. Neurological examination was unremarkable.

**Figure 1.**
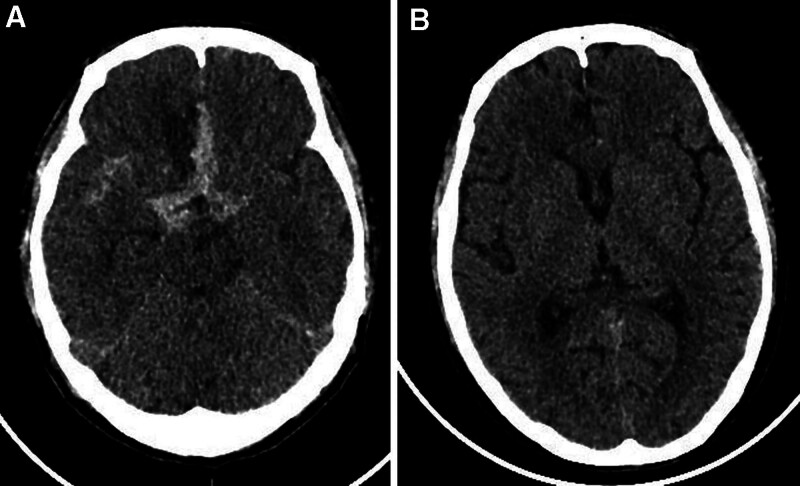
(A) Head CT findings at day 0: a high-density shadow in the cistern of the cerebral longitudinal fissure, cistern of the lateral fossa on both sides, the ambient cistern, and partial cerebral sulci on both sides suggests SAH. (B) Head CT findings at month 2 post-aneurysm treatment. Postoperative changes in the aneurysm can be observed, and the previous high-density shadow in the cistern of the cerebral longitudinal fissure, the right lateral fissure pool, and some brain grooves on both sides have been absorbed. CT = computed tomography, SAH = subarachnoid hemorrhage.

**Figure 2.**
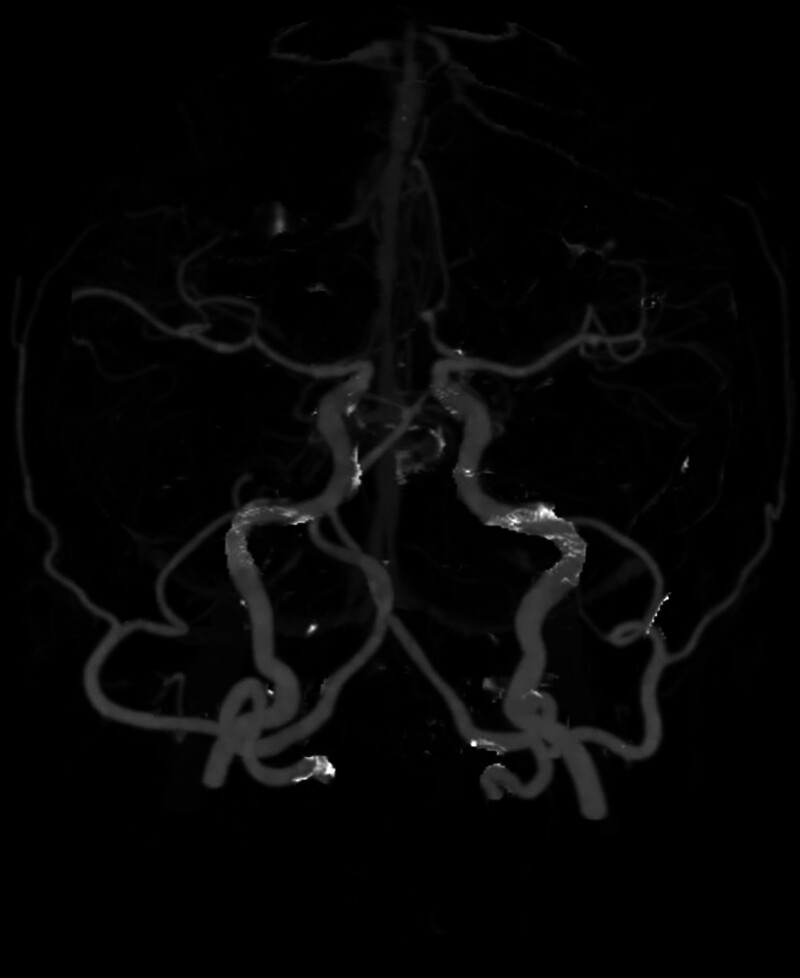
Cranial CTA findings were as follows: a slightly thickened lumen of the anterior communicating artery; a hummocky shadow with smooth margins at the cavernous segment of the right internal carotid artery; variation of the triploid anterior cerebral artery; and a bilateral tortuous V4 segment vertebral artery. Aneurysms cannot be excluded. CTA = computed tomography angiography.

On hospital day 2, the patient reported experiencing dizziness and headache accompanied by nausea but with vomiting or motor impairment. Physical examination revealed an ear temperature of 37.4℃, blood pressure of 164/94 mm Hg, clear consciousness, bilaterally equal and round pupils, and a normal neurological examination. Emergency head CT (Fig. 1A) and CT angiography (Fig. 2) demonstrated subarachnoid hemorrhage (SAH), sinusitis, mild dilatation of the anterior communicating artery, and a suspected aneurysm. The patient was urgently transferred to neurosurgery, where urgent digital subtraction angiography confirmed a ruptured anterior communicating artery aneurysm. Endovascular coiling was subsequently performed with successful occlusion.

**Figure 3.**
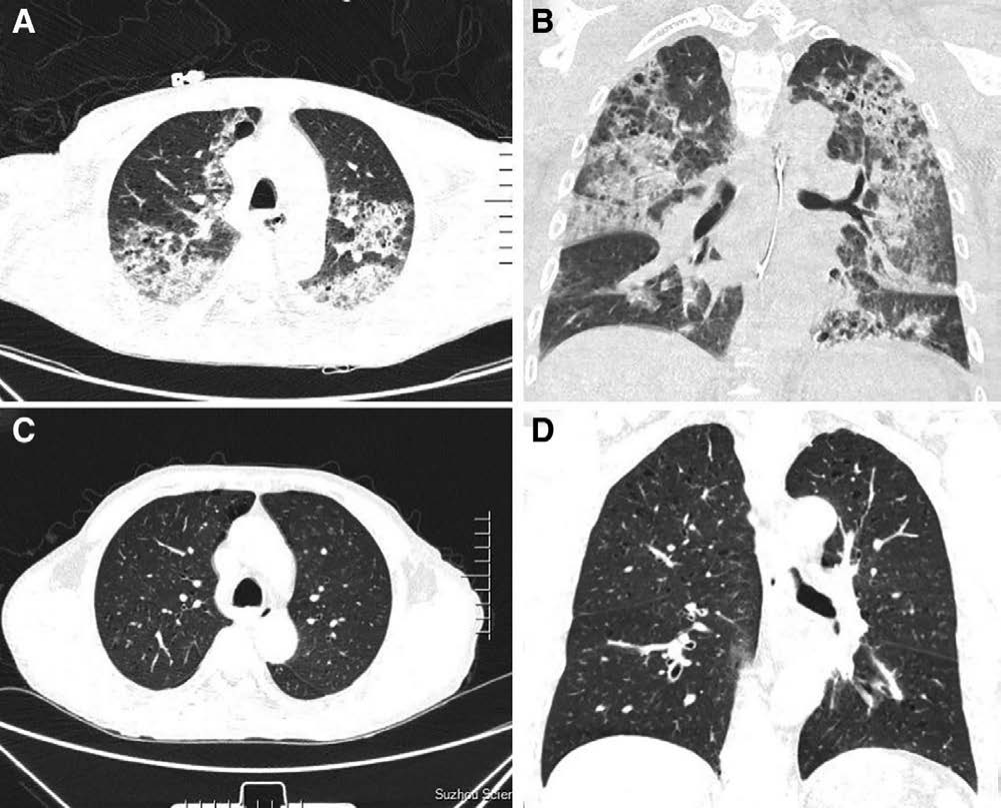
Lung CT findings. (A and B) A grid-like shadow in the anterior inner basal segment of the inferior lobe of the left lung can be seen, suggesting an interstitial change in the inferior lobe of the left lung. (C and D) Slight inflammation is seen, which was significantly more absorbed than before, and both sides of the pleural effusion has been absorbed. CT = computed tomography.

Postoperatively, the patient received intravenous nimodipine, oral clopidogrel, enteric-coated aspirin tablets, and levetiracetam. A follow-up head CT scan showed no rebleeding. The patient’s headache was relieved; however, paroxysmal cough and fever (37.8°C–38.5°C) persisted, and blood-tinged sputum. Repeat chest CT showed multiple patchy consolidations and pleural effusions in both lungs (Fig. 3A and B). Serology was positive for perinuclear antineutrophil cytoplasmic antibodies (p-ANCA) and anti-MPO antibodies, but negative for immunoglobulins, antinuclear antibodies, PR3, and anti-glomerular basement membrane. Laboratory findings: hemoglobin (Hb) was 72 g/L, albumin was 33.0 g/L, and SCr was 485.0 µmol/L. Furthermore, urinalysis showed more than 30 microscopic RBC s/HPF and **2 + **proteinuria. We initiated intravenous methylprednisolone (MP) pulses (240 mg/day for 2 days, followed by 500 mg/day for 3 days) based on the active vasculitis status, after which his SCr level decreased to 416 µmol/L. Continuous intravenous injections of MP (40 mg/day) were then administered along with a single dose of intravenous cyclophosphamide (CTX; 0.6 g). Following treatment, SCr was further reduced to 326 µmol/L, with a urine microscopic RBC at 8 to 10/HPF and urinary protein measurement of 1+. However, 4 days later, his SCr level rose to 372.5 µmol/L with a 24-h urinary protein level of 7194.29 mg. A second MP pulse (240 mg/day IV for 3 days) was administered, which was well-tolerated and resulted in: SCr of 367 µmol/L, 0 RBCs/HPF on urinalysis, and a decrease in the erythrocyte sedimentation rate from 98 to 23 mm/h. Hemoglobin transiently declined to 62 g/L but recovered to 102 g/L. Follow-up head CT demonstrated partial absorption of the SAH. Upon discharge, maintenance therapy consisted of oral prednisone (40 mg/day) and monthly cyclophosphamide infusions (0.6 g).

One month after discharge, the patient returned reporting weakness in both lower limbs. Ultrasound examination of the lower limb vessels conducted at a local hospital revealed thrombosis in the posterior tibial vein and right soleus muscle vein. Furthermore, his D-dimer level was elevated at 33.34 mg/L, SCr level was 427.74 µmol/L, serum albumin was 29.7 g/L, urine microscopic RBC was 0/HPF, urinary protein level was 2+, 24-h urine protein quantification was 3360.21 mg. The patient tested positive for p-ANCA and MPO. Therefore, the patient was promptly readmitted to the Emergency Department of our hospital. Venography of the right lower limb revealed partial filling defects in the deep veins of the right calf, and complete filling defects in the posterior tibial vein. The patient subsequently underwent inferior vena cava filter placement surgery, which resulted in improvements in limb weakness. Standard anticoagulation therapy was initiated, leading to a significant reduction in D-dimer level to 9.73 mg/L by the third day following surgery.

Two months after the cerebral aneurysm coiling, the patient reported no headaches, dizziness, cough, expectoration, blood-tinged sputum, and edema in the eyelids or lower limbs. A follow-up head CT (Fig. 1B) revealed postoperative changes in the aneurysms, and the previous high-density shadow in the cistern of the cerebral longitudinal fissure, right lateral fissure pool, and some brain grooves on both sides had been absorbed. A chest CT scan (Fig. 3C and D) showed slight inflammation, which was significantly more absorbed than before. The patient continued to receive oral prednisone (10 mg/day) alongside intravenous CTX treatment for vasculitis. However, proteinuria continued, as evidenced by 24-h urine protein quantification of 3360.21 mg, urine microscopic RBC of 0/HPF indicating no hematuria, and urinary protein level of 2+. Kidney function remained compromised, with creatinine levels oscillating between 320 and 450 µmol/L, and both p-ANCA/anti-MPO tests remained positive. Due to the patient’s continued use of anticoagulant medication, a kidney biopsy was not performed. The patient remains under close observation.

## 3. Literature review

Our literature review entailed a systematic search of PubMed, Web of Science, Scopus, and China National Knowledge Infrastructure databases, spanning from their inception until February 2024. We utilized keywords or MESH terms such as “antiNeutrophil Cytoplasmic Antibody-Associated Vasculitis,” “eosinophilic granulomatosis with polyangiitis,” “Churg-Strauss,” “microscopic polyangiitis,” and “Subarachnoid Hemorrhage,” without imposing restrictions on the language or geographical origin. We systematically reviewed the literature encompassing all cases of AAV complicated by SAH. After excluding articles that did not meet the requirements and duplicates, 40 articles reporting 41 cases were included. Combined with the present case, a total of 42 cases were analyzed.

The patient characteristics, clinical features, and treatment-related conditions are summarized in Table 1.^[3,9–47]^ Among these cases, 21 patients were diagnosed with EGPA, 10 with GPA, 7 with MPA, and 4 with unclassified AAV. Their ages ranged from 17 to 71 years, with an average age of 48.1 ± 15.7 years. The proportion of female patients was 50.0%. ANCA screening was performed in 37 cases and was positive in 31 (83.78%), with p-ANCA, c-ANCA, and/or MPO-ANCA detected in all positive cases. Most patients presented with peripheral neuropathy, kidney involvement, lung involvement, skin involvement, and other clinical manifestations. Furthermore, 37 of 42 patients were treated with intravenous or oral corticosteroids, 26 received immunosuppressive therapy, including 17 treated with CTX, 3 with rituximab, 1 with cyclosporine A, and 5 with CTX combined with other immunosuppressive agents. The mean follow-up period was 9 months. Patients generally benefited from combined corticosteroids and immunosuppressants. SAH was usually associated with aneurysm rupture.^[16]^ 11 patients (25.58%) had aneurysms. It was reported^[3]^ that AAV with SAH combined with cardiovascular and cerebrovascular events could worsen the prognosis of patients, and the mortality rate was as high as 64.3%.

**Table 1:** Clinical characteristics and treatment of previously reported AAV patients with SAH.

Author	Age/Sex	duration of onset to SAH	Dx	ANCA	Aneurysm	CNS	Treatment for SAH	SI	Steroid	IS agents	Follow-up duration	Outcome
Pokharel A et al,2023^[10]^	67/F	11 d	MPA	ANCA/MPO	+	SAH/ICH	Conservative	Renal/PNS	MP/PRED	RTX	2 wk	Improvement
Palleti SK et al,2023^[11]^	53/F	Present	AAV	MPO	+	SAH	EVD	PNS Renal	PRED	CTX/AZA	4 wk	Significant Improvement
Xie J et al,2022^[3]^	31/M	7 yr	MPA	p-ANCA/MPO	-	SAH	Conservative	PNS/Renal/Pulmonary/	PRED	CTX	6 mo	Significant Improvement
Mino T et al,2022^[9]^	69/F	29 yr	EGPA	–	–	SAH/Cerebral infarctions/Subcortical hemorrhage	Conservative	PNS/Skin	MP	–	1yr	Improvement
Lin RT et al,2021^[12]^	47/F	4 yr	EGPA	–	–	SAH	Conservative	PNS/Skin	MP/PRED	CTX	5 mo	Improvement
Lázaro Romero A et al,2021^[13]^	54/M	3 yr	EGPA	not described	–	SAH/Spinal epidural hematoma	Not specified	PNS/Skin/Asthma	Not specified	Not specified	–	Death
Southam C et al,2019^[14]^	56/M	1 yr	EGPA	p-ANCA/MPO	–	SAH/Spinal SAH/Ventricular hemorrhage	external ventricular drainage	PNS/Renal	MP	–	Not specified	Not specified
Harland TA et al,2019^[15]^	48/F	present	AAV	p-ANCA/MPO	–	SAH	Conservative	Lower extremity weakness with paresthesia	Corticosteroids	RTX	1 wk	Improvement
Ihara K et al,2019^[16]^	85/F	present	MPA	MPO	+	SAH	neck clipping and ventricular drainage	–	MP	–	–	Death from infection
Matsuda S et al,2018^[17]^	48/F	8 mo	EGPA	MPO	+	SAH	Conservative	PNS/Skin/Paresthesia	Betamethasone/MPO	CTX/RTX/AZA	Not specified	Full recovery
Khorolsky C et al,2018^[18]^	40/F	Present	AAV	p-ANCA	+	SAH	coil embolization	PNS/Renal/Pulmonary	MP	CTX/RTX	25 mo	Improvement
Lee MXW et al,2017^[19]^	48/F	1 yr	EGPA	MPO	–	SAH	posterior fossa decompressive and EVD	Mononeuritis multiplex	MP/PRED	CTX	–	Death from intracranial hemorrhage and infective ventriculitis
Aratani S et al,2017^[20]^	54/M	1 mo	MPA	MPO	–	SAH	craniectomy	PNS/Renal/Angina	MP	–	–	Death from SAH
Joshi U et al,2017^[21]^	33/M	present	AAV	c-ANCA	–	SAH	Conservative	PNS/Renal	MP	–	3 mo	Improvement and lost to follow-up
ZHOU Jia-xin et al,2017^[22]^	34/M	9 mo	EGPA	MPO	Not specified	SAH	Conservative	PNS/Arthralgia/Abdominal pain	MP	–	lost to follow-up	Improvement and losing follow-up after discharge
Sylvain L et al,2015^[23]^	43/M	3 yr	EGPA	p-ANCA/MPO	–	SAH	Conservative	Asthma/Myalgia/Arthritis/Peroneal/neuritis	MP	CTX	22 mo	Full recovery
Wang X et al,2015^[24]^	24/M	present	MPA	MPO/p-ANCA	–	SAH	Conservative	Renal	PRED	CTX/LEF/MMF	6 mo	Full recovery
Taormina G et al,2014^[25]^	58/M	7 yr	EGPA	p-ANCA	–	SAH	Conservative	PNS/Renal/Pulmonary/ The supra-aortic vessels	PRED	–	2 mo	Significant Improvement
Ito M et al,2014^[26]^	68/M	6 yr	EGPA	Not specified	–	SAH	Conservative	PNS/Arthritis	Corticosteroids	–	2.5 mo	Significant Improvement
Diamanti L et al,2022^[27]^	31/F	long term	EGPA	p-ANCA	–	Spinal SAH	Conservative	PNS/Asthma/Rhinitis	MP/PRED	RTX	15 mo	Significant Improvement
Menditto VG et al, 2013^[28]^	64/F	6 yr	EGPA	MPO	+	SAH	coil embolization	Asthma/Skin	PRED	CTX	6 mo	Full recovery
Kimura H et al, 2012^[29]^	44/F	3 yr	MPA	MPO	+	SAH	neck clipping	Renal/Pulmonary	MP	CTX	1 yr	Full recovery
Go MH et al, 2012^[30]^	39/M	9 mo	EGPA	MPO	–	SAH/intraventricular hemorrhage	coil embolization	PNS/Renal/Pulmonary/Skin	MP/PRED	CTX	–	Death
Shimizu K et al, 2011^[31]^	60/F	9 yr	EGPA	–	–	SAH	Conservative	phrenic nerve/PNS/Skin	PSL	CsA	3 mo	Improvement
Miles JD et al, 2011^[32]^	74/F	11.5 wk	GPA	c-ANCA/PR3	–	SAH ventricle hemorrhage	Not specified	PNS renal pulmonary skin arthritis liver	MP	CTX	–	Death from SAH
Marnet D et al, 2010^[33]^	63/F	4 yr	GPA	ANCA/PR3	+	SAH	Cerebral aneurysm resection	PNS Renal/Sinusitis/Sinusitis	PRED	CTX/MMF	10 mo	Improvement
Sheerin UM et al,2008^[34]^	37/F	present	EGPA	p-ANCA/MPO	–	SAH	Conservative	-	MP	–	3 mo	Improvement
Mishra S et al,2007^[35]^	45/M	2 yr	EGPA	–	–	ICH, SAH with ventricle hemorrhage	Conservative	Skin/PNS/	MP	CTX	Not specified	Improvement
Fomin S et al,2006^[36]^	17/M	1 yr	GPA	c-ANCA	–	SAH	Conservative	renal/Pulmonary	High-dose steroids	CTX	1 mo	Death from SAH
Sakamoto S et al,2005^[37]^	36/F	8 yr	EGPA	-	+	SAH	coil embolization	Skin/PNS	PRED	–	2 mo	Significant Improvement
Tyvaert L et al,2004^[38]^	47/F	1 mo	EGPA	MPO	–	SAH/Occipital hematoma	Conservative	Skin/PNS	MP	–	Not specified	Improvement
Nardone R et al,2004^[39]^	78/F	Not specified	GPA	c-ANCA	–	SAH/Hemorrhagic infarction	Not specified	PNS Pulmonary/nasal septum/spleen	Not specified	Not specified	–	Death from SAH
Takei H et al,2004^[40]^	34/M	1 yr	GPA	PR3	+	SAH	Aneurysm clipped	PNS/Renal	MP	CTX	Not specified	Improvement
Calvo-Romero JM et al,2002^[41]^	47/F	6 yr	EGPA	MPO	–	SAH	Conservative	Skin/PNS	MP	CTX	2 mo	Full recovery
Cruz DN et al,1997^[42]^	71/M	4 mo	GPA	p-ANCA	–	SAH	Conservative	Renal/Pulmonary/Skin	MP	CTX	6 mo	Significant Improvement
Venning MC et al,1991^[43]^	50/M	4 yr	GPA	c-ANCA	–	SAH	Conservative	Renal/Pulmonary/Skin	PNL	CTX	Not specified	Full recovery
Venning MC et al,1991^[43]^	36/M	6 mo	GPA	-	–	SAH	Conservative	myalgia, arthralgia and resolving orchitis,	PNL	CTX	3 yr	Full recovery
Maloon A et al,1985^[44]^	39/M	3 yr	EGPA	Not specified	–	SAH	Conservative	Pulmonary/Skin	PNL	CTX	4 yr	Death from SAH
DAVID A et al,1963^[45]^	30/M	4 mo	GPA	Not specified	–	SAH/Cerebral infarction	Conservative	PNS	Not specified	Not specified	10 mo	Not improve, Death from arrhythmia
TUHY JE et al,1958^[46]^	39/M	4 mo	GPA	Not specified	–	SAH	Not specified	SKIN	–	–	–	Death
Churg J et al, 1951^[47]^	23/F	24 mo	EGPA	Not specified	Not specified	SAH	Conservative	Renal/Pulmonary/Skin	Not specified	Not specified	Not specified	Death
our case	56/M	2 wk	MPA	p-ANCA/MPO	+	SAH	coil embolization	Pulmonary/	MP/PRED	CTX	2 mo	Improvement

AAV = ANCA-associated vasculitis, ANCA = antineutrophil cytoplasmic antibody, AZA = azathioprine, c-ANCA = cytoplasmic ANCA, CNS = central nervous system, CsA = ciclosporin, CTX = cyclophospham, EGPA = eosinophilic granulomatosis with polyangiitis, EVD = external ventricular drain, GPA = granulomatosis with polyangiitis, IS = immunosuppressive, MMF = mycophenolate mofetil, MP = methylprednisolone, MPA = microscopic polyangiitis, p-ANCA = perinuclear ANCA, PNL = prednisolone, PRED = prednisone, RTX = rituximab.

## 4. Discussion

Clinically, AAV predominantly affects the lungs and kidneys, manifesting with symptoms such as coughing, hemoptysis, hematuria, proteinuria, and potential renal dysfunction. Additionally, it can involve the upper respiratory tract, skin, heart, the nervous system. AAV particularly MPA with MPO-ANCA positivity, is rarely complicated by aneurysmal SAH. This case describes a 56-year-old male with active MPA (manifested by blood-tinged sputum, acute kidney injury, and positive MPO-ANCA) who developed acute SAH due to anterior communicating artery aneurysm rupture, highlighting the complex interplay between systemic vasculitis and cerebrovascular fragility.

MPA associated with aneurysms is very rare, and how it affects blood vessels and leads to aneurysm formation remains unclear. The temporal association between active vasculitis and SAH in this case supports a potential pathogenic link, although definitive causality remains to be established. Several mechanisms may contribute to aneurysm formation and rupture in AAV. First, necrotizing inflammation of the vasa vasorum in medium-sized arteries can disrupt the medial layer, weakening vascular integrity – a process well-documented in AAV-related vascular pathology.^[10,16,20,48]^ Second, neutrophil activation by ANCAs releases proteases such as proteinase 3 and elastase, which induce smooth muscle cell apoptosis and degrade the internal elastic lamina, further predisposing to aneurysm formation.^[40]^ Third, superimposed hypertension during vasculitis flares may exacerbate shear stress on already compromised arterial walls, particularly at bifurcation sites like the anterior communicating artery, where hemodynamic stress is inherently high.^[34]^ Collectively, these factors likely accelerated aneurysm rupture in our patient during active disease. CNS involvement in AAV is uncommon, occurring in 5% to 15% of cases, with ischemic lesions being more prevalent than hemorrhagic complications, especially among Chinese patients, who may exhibit a higher incidence of ischemic CNS manifestations.^[49,50]^ Our patient’s presentation of SAH as the initial CNS manifestation aligns with rare reports of hemorrhagic events in AAV, which are often linked to vasculitic damage to the cerebral vasculature^.[51]^ Notably, headache – observed in our patient – has been identified as a critical warning sign of CNS vascular involvement in AAV, warranting urgent neuroimaging.^[15]^ This underscores the need for heightened vigilance indicating that neurological symptoms in AAV, even in the absence of focal deficits, should prompt immediate evaluation for potentially life-threatening cerebrovascular events.

Management of concurrent AAV and SAH requires balancing urgent neurosurgical intervention with immunosuppressive therapy. The priority for SAH management is to minimize the risk of rebleeding through prompt aneurysm rectification via coil embolization or surgical clipping.^[52]^ The standard induction remission regimen for AAV typically includes corticosteroids along with CTX or RTX.^[53]^ The patient was treated with a treatment combination of corticosteroids and CTX with endovascular coiling, which led to mitigation of SAH symptoms, decreased proteinuria, absence of hemoptysis, and edema, and cessation of cough or blood-streaked sputum, indicating a general improvement in the patient’s health status. Despite these improvements renal function did not exhibit significant improvement. The patient has been on continuous anticoagulant therapy following stent placement in the anterior communicating artery, precluding a kidney biopsy. It has been reported^[54]^ that patients with positive MPO-ANCA have worse kidney outcomes than GPA or EGPA, manifesting as irreversible kidney function and resistance to immunosuppressive therapy. For patients with AAV complicated by renal damage, we should try to achieve early detection, diagnosis, and treatment. In the case of a clinical presentation compatible with small-vessel vasculitis in combination with MPO or PR3-ANCA serologically positive, immunosuppressive therapy should be started immediately, especially in the case of rapid deterioration of renal function, even in the absence of a renal biopsy report. In the 2024 KDIGO AAV management clinical practice guidelines_,_^[55]^ for patients with significantly reduced or rapidly declining glomerular filtration rate (SCr > 354 µmol/L), data supporting the use of rituximab and glucocorticoids are limited. Cyclophosphamide combined with glucocorticoids can be considered, as well as rituximab combined with cyclophosphamide. At the same time, for patients who need dialysis or rapid increase in SCr, plasma exchange can be performed, which can selectively remove autoantibodies and antigen-antibody complexes and improve AAV lesions. In addition, on the premise of excluding contraindications, renal biopsy should be considered as early as possible to understand renal histological changes through renal pathology and better predict renal prognosis. In our case, the patient received pulse methylprednisolone followed by a steroid taper combined with CTX. However, the kidney impairment did not improve, which is consistent with the results of previous reports. To determine whether patients with AAV and CNS damage have worse prognosis, higher relapse rates, or greater treatment resistance compared to those without CNS damage, large-sample, multicenter clinical studies are needed..

Notable limitations include the single-case design and the lack of histopathological confirmation. Persistent questions regarding both the optimal screening protocols for cerebrovascular complications in MPO-ANCA-positive patients and the mechanisms of renal resistance in such cases merit further multicenter investigation.

This study has several important limitations. First, the single-case design inherently limits the generalizability of the findings, necessitating cautious interpretation. Second, histopathological confirmation was unattainable, as a kidney biopsy was contraindicated by the requisite postprocedural anticoagulation therapy; this precludes a definitive correlation between serological markers and the underlying renal pathology. Third, despite a systematic literature review, it is possible that not all relevant cases were identified, which may affect the comprehensiveness of our discussion. Finally, this case underscores persistent questions beyond the scope of a single report, particularly regarding optimal cerebrovascular screening strategies in MPO-ANCA–positive individuals and the mechanisms of renal refractoriness to immunosuppression in such complex cases. These issues highlight the need for larger-scale, multicenter investigations.

## 5. Conclusion

Patients with AAV complicated by SAH represent a rare yet high-risk clinical scenario. Clinicians must recognize that acute neurological symptoms in AAV warrant immediate cerebrovascular evaluation, starting with a non-contrast head CT. MPO-ANCA positivity combined with renal impairment portends a higher risk of SAH among AAV patients, necessitating intensified monitoring. Optimal survival depends on integrated management, including urgent endovascular intervention after diagnosis, along with pulse methylprednisolone (e.g., 500 mg/day for 3 days) combined with induction immunosuppressive therapy using cyclophosphamide or rituximab. Enhanced vigilance for this unique vascular-neurological association in AAV is paramount to improving outcomes.

## Acknowledgments

We thank the Chief Physician, MD, Guo-Cun Hou for raising the attention of this case to be educatable.

## Author contributions

**Conceptualization:** Ping Dong, Jing Li.

**Formal analysis:** Jie Li.

**Investigation:** Ping Dong, Jie Li.

**Supervision:** Jing Li.

**Writing – original draft:** Ping Dong, Jie Li.

**Writing – review & editing:** Jing Li.

## References

[1] Cornec D Cornec-Le Gall E Fervenza FC Specks U. ANCA-associated vasculitis – clinical utility of using ANCA specificity to classify patients. Nat Rev Rheumatol. 2016;12:570–9. 27464484 10.1038/nrrheum.2016.123

[2] Hunter RW Welsh N Farrah TE Gallacher PJ Dhaun N. ANCA associated vasculitis. BMJ. 2020;369:m1070. 32291255 10.1136/bmj.m1070 PMC7179255

[3] Xie J Jia E Wang S. Relapsing subarachnoid hemorrhage as a clinical manifestation in microscopic polyangiitis: a case report and literature review. Clin Rheumatol. 2022;41:3227–35. 35690669 10.1007/s10067-022-06163-6 PMC9485077

[4] Jennette JC Falk RJ Bacon PA. 2012 revised international chapel hill consensus conference nomenclature of vasculitides. Arthritis Rheum. 2013;65:1–11. 23045170 10.1002/art.37715

[5] Geetha D Jefferson JA. ANCA-associated vasculitis: core curriculum 2020. Am J Kidney Dis. 2020;75:124–37. 31358311 10.1053/j.ajkd.2019.04.031

[6] Salvador F. ANCA associated vasculitis. Eur J Intern Med. 2020;74:18–28. 32005600 10.1016/j.ejim.2020.01.011

[7] André R Cottin V Saraux JL; French Vasculitis Study Group (FVSG). Central nervous system involvement in eosinophilic granulomatosis with polyangiitis (Churg-Strauss): report of 26 patients and review of the literature. Autoimmun Rev. 2017;16:963–9. 28709761 10.1016/j.autrev.2017.07.007

[8] De Luna G Terrier B Kaminsky P. Central nervous system involvement of granulomatosis with polyangiitis: clinical-radiological presentation distinguishes different outcomes. Rheumatology (Oxford). 2015;54 :424–32. 25187644 10.1093/rheumatology/keu336

[9] Mino T Sakaguchi H Hasegawa I. Multiple cerebral infarctions accompanied by subcortical and subarachnoid hemorrhaging in bilateral border zone areas in a patient with eosinophilic granulomatosis with polyangiitis. Intern Med. 2022;61:891–5. 34483211 10.2169/internalmedicine.7999-21 PMC8987242

[10] Pokharel A Acharya I Skender J. Unraveling the puzzle: a case report questioning the causal relationship between subarachnoid hemorrhage and microscopic polyangiitis. Cureus. 2023;15:e41088. 37388719 10.7759/cureus.41088 PMC10305980

[11] Palleti SK Larson H Avula S Picken MM Wadhwa A. Subarachnoid hemorrhage in autoimmune vasculitis: a rare presentation of systemic lupus erythematosus-antineutrophil cytoplasmic autoantibody-associated vasculitis overlap syndrome. Cureus. 2023;15:e38482. 37273402 10.7759/cureus.38482 PMC10238284

[12] Lin RT Lin HS Zhang BY Ye LC. [The 484th case:asthma, limb numbness, diplopia, headache]. Zhonghua Nei Ke Za Zhi. 2021;60:179–84. 33503735 10.3760/cma.j.cn112138-20200519-00490

[13] Lázaro Romero A Carilla Sanromán A Horna Cañete L Serrano Ponz M. Spontaneous spinal epidural haematoma and nonaneurysmal subarachnoid haemorrhage in a patient with eosinophilic granulomatosis with polyangiitis. Neurologia (Engl Ed). 2021;36:723–5. 34261616 10.1016/j.nrleng.2020.12.002

[14] Southam C Hahn C. Intracerebral and spinal subarachnoid hemorrhage in eosinophilic polyangiitis. Can J Neurol Sci. 2019;46:475–6. 31179954 10.1017/cjn.2019.63

[15] Harland TA Seinfeld J Cava LF. Anti-neutrophil cytoplasmic antibody associated central nervous system vasculitis with brain and spinal cord subarachnoid hemorrhage: a rare case report and review of the literature. J Clin Neurosci. 2019;62:253–5. 30594448 10.1016/j.jocn.2018.12.001

[16] Ihara K Kimura M Yamamuro M Inoshita S. Microscopic polyangiitis associated with subarachnoid hemorrhage. J Rural Med. 2019;14:125–31. 31191777 10.2185/jrm.2971 PMC6545428

[17] Matsuda S Yoshida S Fujiki Y. Eosinophilic granulomatosis with polyangiitis complicated by subarachnoid hemorrhage and coronary vasculitis: a case report and review of the literature. Rheumatol Int. 2018;38:689–96. 29127573 10.1007/s00296-017-3875-2

[18] Khorolsky C Castellano A Comstock D Brinster NK See SY Garner BF. Systemic lupus erythematosus and antineutrophilic cytoplasmic antibody-associated vasculitis overlap syndrome complicated by subarachnoid hemorrhage: case-based review. Rheumatol Int. 2018;38:2329–35. 30327865 10.1007/s00296-018-4169-z

[19] Lee MXW Teng GG Raju GC Lim AYN. Catastrophic subarachnoid hemorrhage in eosinophilic granulomatosis with polyangiitis without asthma. Int J Rheum Dis. 2017;20:2127–31. 25959920 10.1111/1756-185X.12594

[20] Aratani S Sakai Y Tsuruoka S. A case of microscopic polyangiitis with subarachnoid hemorrhage and cardiovascular complications. J Nippon Med Sch. 2017;84:251–5. 29142188 10.1272/jnms.84.251

[21] Joshi U Subedi R Gajurel BP. Hepatitis B virus induced cytoplasmic antineutrophil cytoplasmic antibody-mediated vasculitis causing subarachnoid hemorrhage, acute transverse myelitis, and nephropathy: a case report. J Med Case Rep. 2017;11:91. 28366165 10.1186/s13256-017-1255-x PMC5376690

[22] Zhou J-X Zhang S-Z Li J. Central nervous system involvement in eosinophilic granulomatosis with polyangiitis: a retrospective study of 8 patients. Chin J Allerg Clin Immunol. 2017;11 :241–6.

[23] Dalia T Parashar S Patel NV Gautam A Dai H Bormann S. Eosinophilic myocarditis demonstrated using cardiac magnetic resonance imaging in a patient with eosinophilic granulomatosis with polyangiitis (churg-strauss disease). Cureus. 2018;10:e2792. 30112268 10.7759/cureus.2792 PMC6089482

[24] Wang X Wang J. Microscopic polyangiitis presenting as spontaneous subarachnoid haemorrhage. Nephrology (Carlton). 2015;20:110. 25329506 10.1111/nep.12352

[25] Taormina G Andolina G Banco MA Costanza-Gaglio EJ Bonura A Buscemi S. An uncommon presentation of eosinophilic granulomatosis with polyangiitis: a case report. J Med Case Rep. 2014;8:190. 24928069 10.1186/1752-1947-8-190 PMC4086703

[26] Ito M Kato N Su CC Kayama T. [A case of Churg-Strauss syndrome with subarachnoid hemorrhage]. Brain Nerve. 2014;66:283–8. 24607952

[27] Diamanti L Berzero G Bini P. Spinal hemorrhage in eosinophilic granulomatosis with polyangiitis (Churg-Strauss). J Neurol. 2014;261:438–40. 24368404 10.1007/s00415-013-7217-3

[28] Menditto VG Di Rienzo A De Nicola M Balzano L Polonara S. Subarachnoid haemorrhage from PICA aneurysm rupture in a Churg-Strauss patient: a case report and a review of the literature. Clin Neurol Neurosurg. 2013;115:197–9. 22683043 10.1016/j.clineuro.2012.04.025

[29] Kimura H Akutsu N Shiomi R Kohmura E. Subarachnoid hemorrhage caused by ruptured intracranial fusiform aneurysm associated with microscopic polyangiitis. Neurol Med Chir (Tokyo). 2012;52:495–8. 22850498 10.2176/nmc.52.495

[30] Go MH Park JU Kang JG Lim YC. Subarachnoid and intracerebral hemorrhage in patients with churg-strauss syndrome: two case reports. J Cerebrovasc Endovasc Neurosurg. 2012;14:255–61. 23210058 10.7461/jcen.2012.14.3.255 PMC3491225

[31] Shimizu K Ohoba H Shimada H Inoue Y Jinn Y Yoshimura N. [A case of Churg-Strauss syndrome with subarachnoid hemorrhage and left phrenic nerve paralysis]. Nihon Kokyuki Gakkai Zasshi. 2011;49:642–6. 22073608

[32] Miles JD McWilliams L Liu W Preston DC. Subarachnoid hemorrhage in wegener granulomatosis: a case report and review of the literature. CNS Spectr. 2011;16:121–6. 24725388 10.1017/S1092852912000284

[33] Marnet D Ginguené C Marcos A. [Wegener granulomatosis and aneurysmal subarachnoid hemorrhage: an insignificant association?]. Neurochirurgie. 2010;56:331–6. 20451938 10.1016/j.neuchi.2010.04.001

[34] Sheerin UM Barreto J Brown MM Brew S Losseff NA. Subarachnoid haemorrhage as the first clinical manifestation of Churg-Strauss syndrome. J Neurol. 2008;255:783. 27517590 10.1007/s00415-008-0975-7

[35] Mishra S Das CP Das A Prabhakar S. Intracerebral hemorrhage in a patient with Churg-Strauss syndrome. Neurol India. 2007;55:416–8. 10.4103/0028-3886.37102 18040124

[36] Fomin S Patel S Alcasid N Tang X Frank E. Recurrent subarachnoid hemorrhage in a 17 year old with wegener granulomatosis. J Clin Rheumatol. 2006;12:212–3. 10.1097/01.rhu.0000231369.89028.36 16891931

[37] Sakamoto S Ohba S Eguchi K. Churg-Strauss syndrome presenting with subarachnoid hemorrhage from ruptured dissecting aneurysm of the intracranial vertebral artery. Clin Neurol Neurosurg. 2005;107:428–31. 16023541 10.1016/j.clineuro.2004.09.020

[38] Tyvaert L Devos P Deloizy M Belhadia A Stekelorom T. [Peripheral and central neurological manifestations in a case of Churg Strauss syndrome]. Rev Neurol (Paris). 2004;160:89–92. 14978400 10.1016/s0035-3787(04)70853-x

[39] Nardone R Lochner P Tezzon F. Wegener’s granulomatosis presenting with intracerebral hemorrhages. Cerebrovasc Dis. 2004;17:81–2. 10.1159/000073905 14534381

[40] Takei H Komaba Y Kitamura H. Aneurysmal subarachnoid hemorrhage in a patient with Wegener’s granulomatosis. Clin Exp Nephrol. 2004;8:274–8. 15480908 10.1007/s10157-004-0280-4

[41] Calvo-Romero JM del Carmen Bonilla-Gracia M Bureo-Dacal P. Churg-Strauss syndrome presenting as spontaneous subarachnoid haemorrhage. Clin Rheumatol. 2002;21:261–3. 12111635 10.1007/s10067-002-8293-4

[42] Cruz DN Segal AS. A patient with Wegener’s granulomatosis presenting with a subarachnoid hemorrhage: case report and review of CNS disease associated with Wegener’s granulomatosis. Am J Nephrol. 1997;17:181–6. 9096451 10.1159/000169095

[43] Venning MC Burn DJ Bashir SH Deopujari CE Mendelow AD. Subarachnoid haemorrhage in Wegener’s granulomatosis, with negative four vessel angiography. Br J Neurosurg. 1991;5:195–8. 1863381 10.3109/02688699108998467

[44] Maloon A Fritz VU Kaplan CL. Neurological complications of systemic vasculitis. A report of 2 cases. S Afr Med J. 1985;68:603–5. 2864747

[45] Drachman DA. Neurological complications of Wegener's granulomatosis. Arch Neurol. 1963;8 :145–55.

[46] Tuhy JE Maurice GL Niles NR. Wegener’s granulomatosis. Am J Med. 1958;25:638–46. 13582971 10.1016/0002-9343(58)90052-4

[47] Churg J Strauss L. Allergic granulomatosis, allergic angiitis and periarthritis nodosa. Am J Pathol. 1951;27:277–301. 14819261 PMC1937314

[48] Mavrogeni S Manoussakis MN Karagiorga TC. Detection of coronary artery lesions and myocardial necrosis by magnetic resonance in systemic necrotizing vasculitides. Arthritis Rheum. 2009;61:1121–9. 19644909 10.1002/art.24695

[49] Zheng Y Zhang Y Cai M Lai N Chen Z Ding M. Central nervous system involvement in ANCA-associated vasculitis: what neurologists need to know. Front Neurol. 2018;9:1166. 30687221 10.3389/fneur.2018.01166 PMC6335277

[50] Ma TT Li ZY Geng YS Chen M Zhao MH. Central nervous system involvement in patients with antineutrophil cytoplasmic antibody-associated vasculitis: a study of 29 cases in a single Chinese center. Clin Rheumatol. 2020;39:2185–93. 32062770 10.1007/s10067-020-04975-y

[51] Sherri A Mortada MM Makowska J Lewandowska-Polak A. Primary angiitis of the CNS and ANCA-associated vasculitis: from pathology to treatment. Rheumatol Int. 2024;44:211–22. 37777632 10.1007/s00296-023-05461-9 PMC10796583

[52] Claassen J Park S. Spontaneous subarachnoid haemorrhage. Lancet. 2022;400:846–62. 35985353 10.1016/S0140-6736(22)00938-2 PMC9987649

[53] Robson JC Grayson PC Ponte C; DCVAS Study Group. 2022 American College of Rheumatology/European alliance of associations for rheumatology classification criteria for granulomatosis with polyangiitis. Arthritis Rheumatol. 2022;74:393–9. 35106964 10.1002/art.41986

[54] Geetha D Jin Q Scott J. Comparisons of guidelines and recommendations on managing antineutrophil cytoplasmic antibody-associated vasculitis. Kidney Int Rep. 2018;3:1039–49. 30197970 10.1016/j.ekir.2018.05.007 PMC6127414

[55] Kidney Disease: Improving Global Outcomes (KDIGO) ANCA Vasculitis Work Group. KDIGO 2024 clinical practice guideline for the management of antineutrophil cytoplasmic antibody (ANCA)-associated vasculitis. Kidney Int. 2024;105:S71–S116. 38388102 10.1016/j.kint.2023.10.008

